# Diagnostic Performance and Usability of the Genedrive^®^ HCV ID Kit in Two Decentralized Settings in Cameroon and Georgia

**DOI:** 10.3390/diagnostics11050746

**Published:** 2021-04-22

**Authors:** Francois M. J. Lamoury, Richard Njouom, Marie Amougou-Atsama, Euloge Yiagnigni Mfopou, Nino Berishvili, Manana Sologashvili, Emmanuel Fajardo, Agnes Malobela, Aurélien Macé, Maxwell Chirehwa, Maia Alkhazashvili, Elena Ivanova Reipold

**Affiliations:** 1 Foundation for Innovative New Diagnostics, 1202 Geneva, Switzerland; lamoury_fr@hotmail.com (F.M.J.L.); fajardoe@who.int (E.F.); Agnes.Malobela@finddx.org (A.M.); Aurelien.Mace@finddx.org (A.M.); 2 Centre Pasteur du Cameroun, Yaoundé 1274, Cameroon; njouom@pasteur-yaounde.org (R.N.); marieamougou164@yahoo.com (M.A.-A.); 3 Polyclinique Les Promoteurs de la Bonne Santé, Yaoundé 8463, Cameroon; eyiagni70@gmail.com; 4 Lugar Center for Public Health Research, National Center for Disease Control and Public Health, 0198 Tbilisi, Georgia; n.berishvili@ncdc.ge (N.B.); M.Alkhazashvili@ncdc.ge (M.A.); 5 Hepa Plus, 0190 Tbilisi, Georgia; mananasolog@yahoo.com; 6 Division of Clinical Pharmacology, University of Cape Town, Cape Town 7700, South Africa; mtchirehwa@gmail.com

**Keywords:** Hepatitis C, HCV RNA, diagnostics, point-of-care, Genedrive

## Abstract

Point-of-care diagnostics have the potential to increase diagnosis and linkage to care and help reach the WHO targets to eliminate hepatitis C virus (HCV) by 2030. Here, we evaluated the diagnostic accuracy of Genedrive HCV ID assay for the qualitative detection of HCV RNA in decentralized settings in two low- and middle-income countries using fresh plasma specimens from 426 participants. The Abbott RealTime HCV assay was used as the gold standard. Genedrive HCV ID assay was conducted by different users. Users also completed questionnaires to assess the usability of Genedrive. At detection thresholds of 12 IU/mL or 30 IU/mL, 1000 IU/mL, and 2362 IU/mL, the sensitivity was 96.2% (95% CI: 92.7–98.4), 100% (98.2–100), and 100% (98.2–100), respectively; the specificity was 99.5% (95% CI: 97.4–100), 99.5% (97.5–100), and 98.7% (96.1–100), respectively. All genotypes detected using the gold-standard assay were also detected with Genedrive. Users found Genedrive easy to use. Genedrive is a simple and accurate test to confirm chronic HCV infection in decentralized, real-life, resource-limited settings. This novel diagnostic tool could contribute to closing the current gap in HCV diagnosis.

## 1. Background

Hepatitis C virus (HCV) infection can lead to chronic disease that may progress for decades without being noticed until symptoms of advanced liver disease appear [1]. Approximately 71 million people worldwide are living with chronic HCV infection, and more than 80% of them are living in low- and middle-income countries (LMICs) [2]. The World Health Organization (WHO) has called for the elimination of HCV by 2030 [3]. Treatment for HCV is becoming more widely available worldwide, with the development of short-course oral direct-acting antiviral (DAA) regimens with higher tolerability and cure rates and the introduction of generic formulations [4,5,6]. However, access to treatment remains limited due to insufficient diagnostic services and poor linkage to care.

Nucleic acid testing for quantitative HCV RNA determination is mainly performed using high-throughput platforms in specialized laboratories. The cost of these assays is generally high, and turnaround times for results to reach patients can be several weeks in duration. More portable, affordable, and easy-to-use platforms that can be used in district hospitals or clinics may help decentralize and increase access to HCV testing in LMICs [7].

One HCV RNA assay that requires fewer technical skills to operate is the Genedrive^®^ HCV ID Kit (Genedrive Diagnostics Ltd, Manchester, UK), henceforth referred to as Genedrive. This test meets most of the technical requirements for a virological test, as defined in the target product profile developed by FIND (Foundation for Innovative New Diagnostics) and WHO [8]. Genedrive detects HCV RNA in a small volume of plasma (30 µL) using reverse transcription polymerase chain reaction (RT-PCR). The system provides a simple, qualitative result without the need for specialist knowledge or data interpretation [9]. The Genedrive instrument is small and can be easily operated in a range of decentralized laboratory settings with limited requirements for ancillary equipment or test materials. The Genedrive HCV ID Kit comprises lyophilized PCR reagents packaged into a single-use, disposable cartridge, and the testing procedure consists of 12 manual steps [10]. The test turnaround time is about 90 min. It is intended to be a confirmatory test of current HCV infection following a positive HCV antibody test. The test is CE-marked [11] and WHO prequalified [12]. One study found Genedrive to have an analytical sensitivity of 2362 IU/mL, with 98.6% sensitivity and 100% specificity [13]. However, the performance was evaluated on leftover frozen plasma samples obtained from a research laboratory in Europe. The performance of Genedrive with freshly collected samples in real-world LMIC settings, operated by intended users, has not yet been assessed.

Genedrive has the potential to simplify testing requirements and decentralize HCV RNA testing for the confirmation of HCV viremia. The aim of this study was to evaluate the diagnostic performance and usability of Genedrive in laboratory-based real-life settings in Cameroon and Georgia.

## 2. Methods

### 2.1. Study Design

This cross-sectional study was performed and reported in accordance with the Standards for Reporting of Diagnostic Accuracy Studies (STARD) guidelines [14]. The study was conducted in small laboratories in primary healthcare settings where, typically, HCV RNA testing is not available. Informed consent was obtained from participants in Georgia and Cameroon, who were then prospectively enrolled in the study. The study protocol was approved by the respective local ethics committees in Cameroon (N°2019/06/1166/CE/CNERSH/SP, 14 June 2019) and Georgia (N°8160-2/1, 1 December 2018). The diagnostic performance of Genedrive (index test) was evaluated against a gold standard (reference test), the Abbott RealTime HCV viral load assay (Abbott Molecular Inc., Des Plaines, IL, USA), henceforth referred to as Abbott HCV.

### 2.2. Study Population and Study Settings

Individuals who fulfilled the eligibility criteria were invited to participate in the study until the desired sample size was reached. Recruitment took place between June and October 2019. Three population groups were considered: “HCV risk,” “HCV seropositive,” and “HCV treatment.” The “HCV risk” group included individuals at risk of having HCV infection based on past and/or current exposure to risk factors, as defined in the WHO [15] and Centers for Disease Control and Prevention (CDC) guidelines [16]. The “HCV seropositive” group included individuals with a documented positive HCV serology result. Finally, the “HCV treatment” group included individuals diagnosed with chronic HCV infection who initiated or completed a course of DAA therapy and who presented at the clinical site for treatment monitoring or test of cure (i.e., sustained virological response).

Two sites per country were used for testing, one site for the index test and another for the reference test. In Georgia, recruitment and index testing was done at a Hepa Plus harm reduction site in Tbilisi, and reference testing was done at the National Center for Disease Control (NCDC). In Cameroon, recruitment and reference testing was conducted at the Centre Pasteur of Cameroon (CPC) in Yaoundé, and index testing was performed at the clinic “les Promoteurs de la Bonne Santé (PBS),” also in Yaoundé. Neither of the country index testing facilities had any prior experience in PCR-based assays and were blind to the results of reference testing. At both sites, reference and index testing were carried out by different users, and no data were shared.

### 2.3. Testing Methods

Participants were asked to provide 8 mL of venous blood, collected in standard K_2_ EDTA tubes. Plasma was aliquoted within 6 h of blood collection, and Genedrive testing carried out within 24 h, using 30 μL of plasma. The Genedrive testing procedure consisted of 12 steps, from sample input to result reporting. One plasma aliquot was used for testing with the Abbott HCV within 4 weeks of blood collection, using the m2000sp/m2000rt platform. Another aliquot of plasma was used for HCV genotype analysis, performed using Sanger sequencing in Cameroon and the Abbott RealTime HCV Genotype II Assay [17] in Georgia. The remaining aliquots were stored at −80 °C and used for repeat testing and resolution of discrepant results. All testing procedures were performed in accordance with the manufacturers’ instructions.

### 2.4. Usability of Genedrive

All users received training on Genedrive from the manufacturer prior to the start of the studies. Two structured questionnaires, capturing ease-of-use, level of training received, problems encountered, and overall opinion of the technology were completed by each user based on their experience of the test system. Answers were rated using a 5-point Likert scale, from strongly agree to strongly disagree (Supplementary Material Table S1).

### 2.5. Statistical Analysis

Assuming a sensitivity and specificity of 95% and 97.5%, respectively, a power of 80%, and a significance level of 5%, it was calculated that a minimum sample size of 200 participants with detectable HCV RNA and 200 participants with no detectable HCV RNA was required.

The results of Genedrive were compared with those of the Abbott HCV to calculate sensitivity and specificity, along with 95% confidence intervals (CI). Sensitivity and specificity were calculated using different detection thresholds with the reference assay, namely: 30 IU/mL in Georgia [18] and 12 IU/mL in Cameroon; 1000 IU/mL based on recommendations from the European Association for the Study of the Liver (EASL) [19] and 2362 IU/mL based on Genedrive’s lower limit of detection.

The rate of invalid results was estimated by calculating the total number of tests without a positive or negative result given by the Genedrive instrument, either because of an indeterminate result or a failed control. Invalid results were excluded from the diagnostic accuracy analyses. Descriptive statistics were used to describe the study population. All analyses were performed using the statistical software R (R Foundation for Statistical Computing, version 3.6).

## 3. Results

### 3.1. Characteristics of the Study Population

**Figure 1:**
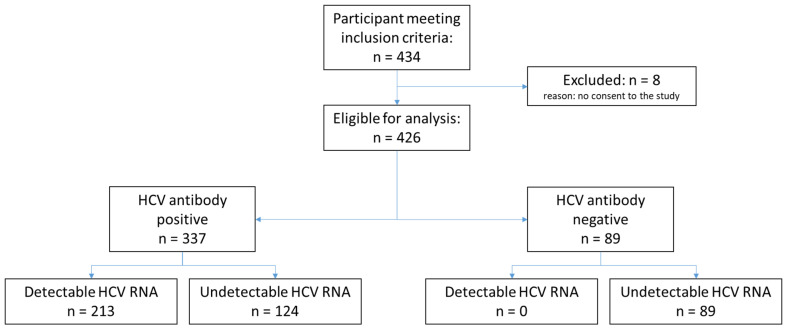
Study flow diagram.

Among the 434 participants who met the inclusion criteria, 8 participants were excluded due to lack of consent, resulting in a total of 426 enrolled participants. The total number of participants classified according to HCV antibody status and HCV RNA is shown in Figure 1. The demographic characteristics of the participants are displayed in Table 1. Among all enrolled participants, the median age was 47 years, and 73.9% were male. In Georgia, 99.6% of participants had a history of injecting non-prescription drugs compared to none in Cameroon. Overall, 1.3% of participants in Cameroon were HIV-positive versus 1.1% in Georgia. Only 11.0% of participants had received HCV treatment, all from Georgia. Overall, 50.0% of participants had detectable HCV RNA levels. Of the samples with detectable HCV RNA, 93% had genotype data available, with genotype 1 being the most common (52.7%).

### 3.2. Genedrive Diagnostic Performance

The sensitivity and specificity results at different detection thresholds using the Abbott HCV reference test are shown in Table 2. Using a detection threshold of 12 or 30 IU/mL, the sensitivity and specificity of the Genedrive were 96.2% (95% CI: 92.7–98.4) and 99.5% (95% CI: 97.4–100). There were eight false-negative results using Genedrive; the highest viral load among these samples was 98 IU/mL with the reference test. There was one false-positive result; this sample was from a Georgian participant in an HCV risk group who had received a previous HCV seronegative result. As anticipated, the sensitivity of Genedrive increased to 100% (95% CI: 98.2–100) using the higher detection thresholds of 1000 and 2362 IU/mL. The specificity remained the same with the 1000 IU/mL threshold but decreased slightly to 98.7% (95% CI: 96.1–99.7) with the 2362 IU/mL threshold, owing to three false-positive results.

In this study, all specimens that could be genotyped (with HCV viral loads >1000 IU/mL) had concordant results between Genedrive and Abbott HCV, showing a sensitivity of 100% (95% CI: 98.2–100) across all HCV genotypes tested. Among 426 plasma samples undergoing Genedrive testing, two were found to be “indeterminate” and five “control failed.” When repeated with remnant plasma samples, all seven tests were rendered valid. The overall invalid test rate was, therefore, 1.6% (1.9% in Cameroon and 1.5% in Georgia) (Table 3).

### 3.3. Genedrive Usability

**Figure 2:**
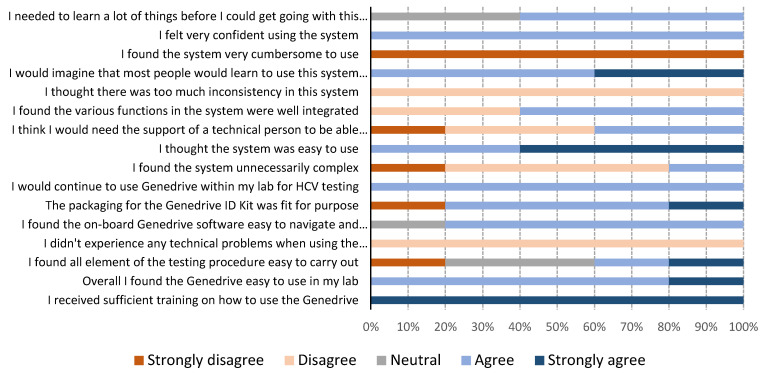
Genedrive system usability results from five users.

The results of the usability questionnaires are shown in Figure 2. Genedrive users in Georgia were two nurses who were not familiar with performing laboratory testing. In Cameroon, the users were three laboratory technicians who were not skilled in molecular testing, but who were familiar with general laboratory techniques. All users reported they found the Genedrive system easy to use. One user mentioned a need for initial training, stating that “training is really necessary before using the machine; it is true the instructions are clear but technical assistance is required for the first use.” All users strongly agreed with the statement that they received sufficient training on how to use the Genedrive. Answers relating to the ease-of-use of carrying out the 12-step testing procedure varied, with two users agreeing it was easy, one user strongly disagreed, and two had neutral views. The most frequently encountered issues were related to the optional printer, either due to the paper rolling up and jamming the printer or because of the connector to the instrument, which was loose and, therefore, easily disconnected from the printer. Users commented “we had the label rolling on itself when printing and it was not easy to remove,” and “power cords for the printers are not holding very well, sometimes we have to hold them before printing.”

## 4. Discussion

This is the first field evaluation to date that has assessed the diagnostic accuracy and usability of Genedrive in a large, real-life cohort in different decentralized laboratories located in LMICs with staff who had no previous experience in molecular testing. Decentralized settings such as harm reduction sites or peripheral clinics with small laboratories are the intended settings where Genedrive could be deployed. This is also the first study using Genedrive to test a large number of fresh specimens with different HCV genotypes. Previous evaluations of Genedrive have been conducted using panels of frozen plasma samples in reference laboratories [13,20,21]. The first clinical validation study of Genedrive included “real-life clinical settings,” with 130 plasma and serum samples collected from routine HCV RNA diagnostic testing across 10 African countries; however, these samples were frozen and shipped to be tested in South Africa by experienced laboratory personnel [13].

Our results for sensitivity using the 12 or 30 IU/mL detection thresholds were slightly lower than those of previous studies, owing to eight samples (3.8%) generating false-negative results; the highest RNA level in these samples was 98 IU/mL, using the reference test. In previous studies, the number of false-negative results with Genedrive was lower: 1.4% [13] and 0% [21], both in India. The majority of false-negative results in these studies had viral loads <1000 IU/mL. The specificity found in our study of 99.5% (95% CI: 97.4–100) was slightly lower than that found in previous studies, which all reported a specificity of 100%. This was due to one sample with undetectable HCV RNA using reference testing and HCV seronegative that was tested positive with Genedrive. The testing of this discordant sample was not repeated due to an insufficient volume. Our findings also corroborate previous research demonstrating the ability of Genedrive to identify all major HCV genotypes.

The invalid test rate in our study was 1.6%, lower than the 3.1% reported in an evaluation study by WHO [20], which is a positive finding for the use of Genedrive in LMICs by staff with no prior molecular testing experience. Consistent with this, users in both Cameroon and Georgia found the Genedrive easy to use, as indicated by their responses to the usability questionnaire. Most of the issues they identified were related to the printer and its occasional disconnection from the instrument due to the use of USB adapters.

One of the key benefits of Genedrive is the small volume of plasma needed for analysis (30 μL); with the rapid development of new plasma separation devices, it should be possible to obtain this volume of plasma from finger-stick blood samples, obviating the need for centrifugation [22]. The test does not contain any hazardous chemicals, such as guanidinium thiocyanate, and with the small volume of reagent used (approximatively 135 μL), the test does not require any specific disposal measures other than standard biohazard waste disposal procedures. This could be an important advantage in decentralized LMIC settings, where access to high-temperature incinerators to dispose of toxic reagents can be problematic [23].

The Genedrive instrument has a small footprint, however, it requires an electrical power supply, thus limiting its placement to facilities equipped with the necessary electrical set up, which may exclude the device from being used at a lower level health facility not equipped with necessary wiring. Future developments will be required to equip the instrument with the battery, enabling several tests to be performed without the need for an uninterruptible power supply in settings where power cuts are commonplace.

Another technology with a small footprint that is already available on the market is the GeneXpert platform (Cepheid, Sunnyvale, CA, USA), which uses a self-contained cartridge for the quantitative measurement of HCV RNA [24]. The advantage of GeneXpert is its capability to run a wide range of disease-specific tests [25,26], whereas the Genedrive is currently limited to testing for HCV, mitochondrially encoded 12S RRNA (MT-RNR1), and severe acute respiratory syndrome coronavirus 2 (SARS-CoV-2). GeneXpert HCV VL Fingerstick test also has a smaller number of manual steps and a shorter turnaround time. The Genedrive HCV ID Kit costs between USD $25 and $30, including shipping costs, import duties, and distributor margins [27], which is slightly higher than that of the Xpert HCV viral load assay (USD $21.64) [28]. The cost of the Genedrive instrument is about USD $5000, making it more affordable than the one-module configuration GeneXpert Edge (USD $8495). The choice of platform, therefore, depends on the type of setting and whether integration of diagnostic services is required.

The analytical sensitivity of Genedrive HCV assay is 2362 IU/mL using 30 µL of plasma, which is lower than that of the Xpert HCV viral load (10 IU/mL using 1 mL plasma) and Xpert HCV VL Fingerstick test (100 IU/mL using 100 μL capillary blood) [29]. However, the optimal limit of detection for an HCV point-of-care test to diagnose 97% of HCV-infected people is 1318 IU/mL, as determined by an analysis of a large, global dataset [30]. According to this dataset published by Freeman et al. [30], the limit of detection of 3000 IU/mL will allow detecting at least 95% of all HCV RNA-positive cases.

Currently, the need for plasma for Genedrive testing requires centrifugation of EDTA whole blood, which necessitates a basic laboratory setting to perform HCV testing. The future use of direct capillary blood would greatly enhance the use of Genedrive in remote settings, particularly in settings such as services for people who inject drugs, where individuals could be tested using a finger-stick blood sample [31]. Notwithstanding its limitations, the arrival of Genedrive on the market is expected to bolster competition and contribute to closing the current diagnostic gap in HCV diagnosis.

Decentralization of testing through point-of-care testing has the potential to support increased access to HCV diagnostics and improve linkage to care [32]. Indeed, many countries implementing HCV elimination programs experienced difficulties in identifying infected individuals, following an initial phase of treatment scale-up with pre-identified patients [33]. Near point-of-care solutions like Genedrive could support case-finding activities in decentralized settings; however, further implementation research is needed to confirm these assumptions.

This study has several limitations. First, the performance of Genedrive was assessed for diagnostic purposes, with most participants being treatment-naïve and only 40 participants who had received treatment in Cameroon were included in the study. Therefore, the performance of Genedrive to assess sustained virological response (SVR) could not be determined because most participants had undetectable viral loads. It is important to note that the GeneDrive HCV ID assay is not intended to be used as a test of cure. A second limitation of the study is the difference in recruitment and Genedrive testing between the sites. In Georgia, the recruitment and Genedrive testing were performed at a harm reduction site, and the plasma aliquots were then sent to a reference laboratory. In Cameroon, the recruitment and reference testing were both carried out at a reference center, and then the plasma samples were sent to a private clinic for Genedrive testing. Although the transport of plasma samples to the Genedrive testing site in Cameroon took place under cold-chain conditions, we cannot exclude the possibility that this transportation may have impacted the accuracy of the test, as the sensitivity was slightly lower in Cameroon than in Georgia: 92.4% (95% CI: 84.9–96.8) versus 99.2% (95% CI: 95.5–99.9). Finally, the usability of Genedrive was only assessed among a small number of users. Although most answers were consistent across users, answers to the questions relating to the testing procedure varied. Therefore, future studies involving a larger number of users will be necessary to validate our findings.

## 5. Conclusions

In conclusion, this study contributes additional evidence of the potential of Genedrive. It can be used as a test for the confirmation of HCV viremia following a positive antibody test in decentralized settings in LMICs. Furthermore, Genedrive can be successfully used by staff with no previous experience in molecular testing.

## Figures and Tables

**Table 1:** Characteristics of enrolled participants by site.

Characteristics	Cameroon (*n* = 156)	Georgia (*n* = 270)	Total (*n* = 426)
Female	93 (59.6)	18 (6.7)	111 (26.1)
Male	63 (40.4)	252 (93.3)	315 (73.9)
Median age (range), years	63 (21–83)	43 (18–69)	47 (18–83)
Positive HCV antibody	156 (100)	181 (67.0)	337 (79.1)
Positive HIV antibody	2 (1.3)	3 (1.1)	5 (1.2)
HCV-positive mother	3 (1.9)	0 (0.0)	3 (0.7)
Injects non-prescription drugs	0 (0.0)	269 (99.6)	269 (63.1)
Treated in past 12 months	47 (30.1)	0 (0.0)	47 (11.0)
Abbott HCV RNA undetectable	64 (41.0)	149 (55.0)	213 (50.0)
Abbott HCV RNA detectable	92 (59.0)	121 (45.0)	213 (50.0)
HCV genotype determined *	85 (41.5)	120 (58.5)	205 (96.2)
Genotype 1 ^†^	33 (38.8)	75 (62.5)	108 (52.7)
Genotype 2 ^†^	13 (15.3)	18 (15.0)	31 (15.1)
Genotype 3 ^†^	0 (0.0)	21 (17.5)	21 (10.2)
Genotype 4 ^†^	38 (44.7)	0 (0.0)	38 (18.5)
Genotype undetermined ^†^	1 (1.2)	6 (5.0)	7 (3.4)

Data are number (%) unless otherwise indicated. HCV: Hepatitis C virus; HIV: Human immunodeficiency virus. * The denominator is the number of samples with HCV RNA detected by Abbott HCV RealTime assay. ^†^ The denominator is the number of HCV genotypes determined.

**Table 2:** Overall sensitivity and specificity of the Genedrive^®^ HCV ID Kit compared with the sensitivity and specificity of the Abbott RealTime HCV Viral Load Assay at different detection thresholds.

		Abbott: 12–30 IU/mL Threshold		Total	Diagnostic Accuracy (95% CI)
		Target Detected	Target Undetected		
Genedrive^®^ HCV ID assay	Positive	205	1	206	**Sensitivity:** 96.2% (92.7–98.4) **Specificity:** 99.5% (97.4–100)
	Negative	8	212	220	
	Total	213	213	426	
		**Abbott: 1000 IU/mL threshold**		Total	
		Target detected	Target undetected		
Genedrive^®^ HCV ID assay	Positive	205	1	206	**Sensitivity:** 100% (98.2–100) **Specificity:** 99.5% (97.5–100)
	Negative	0	220	220	
	Total	205	221	426	
		**Abbott: 2362 IU/mL threshold**		Total	
		Target detected	Target undetected		
Genedrive^®^ HCV ID assay	Positive	203	3	206	**Sensitivity:** 100% (98.2–100) **Specificity:** 98.7 (96.1–99.7)
	Negative	0	220	220	
	Total	203	223	426	

Abbreviations: IU: International Units; CI: Confidence interval.

**Table 3:** Invalid test rate for Genedrive^®^ HCV ID Kit by site.

Genedrive Result	Cameroon	Georgia	Total
Negative	69 (44.2)	146 (54.1)	215 (50.5)
Positive	84 (53.8)	120 (44.4)	204 (47.9)
Indeterminate	2 (1.3)	0 (0.0)	2 (0.5)
Control failed	1 (0.6)	4 (1.5)	5 (1.2)
Total tests	156 (36.6)	270 (63.4)	426 (100)
Invalid tests repeated	3 (1.9)	4 (1.5)	7 (1.7)
Negative	2 (66.7)	3 (75.0)	5 (71.4)
Positive	1 (33.3)	1 (25.0)	2 (28.6)
Total tests performed	159 (36.7)	274 (63.3)	433 (100)
Total invalid rate	3 (1.9)	4 (1.5)	7 (1.6)

Data are number (%) unless otherwise indicated.

## Data Availability

The data presented in this study are available on request from the corresponding author. Some human data are not publicly available due to data protection reasons.

## Supplementary Materials

The following are available online at https://www.mdpi.com/article/10.3390/diagnostics11050746/s1, Table S1: Detailed Genedrive System Usability Scale Questionnaire.
