# Supplementary material for: Diagnostic Performance and Usability of the Genedrive® HCV ID Kit in Two Decentralized Settings in Cameroon and Georgia
Source: Diagnostics (Basel). 2021 Apr 22;11(5):746. doi: 10.3390/diagnostics11050746 (PMC8143533; doi:10.3390/diagnostics11050746)
Supplement: Supplementary file 1 [file diagnostics-11-00746-s001.zip › diagnostics-1148495-supplementary.pdf]

Supplementary Material Table S1: Detailed Genedrive System Usability Scale Questionnaire

Questionnaire part 1: Genedrive® System Usability Scale designed by Genedrive

1. ‘I received sufficient training on how to use the Genedrive® and Genedrive® HCV ID Kit.’

	Strongly Disagree	Disagree	Neutral	Agree	Strongly agree
CMR A	□	□	□	□	■
CMR B	□	□	□	□	■
CMR C	□	□	□	□	■
GEO A	□	□	□	□	■
GEO B	□	□	□	□	■

2. ‘Overall, I found the Genedrive® and Genedrive® HCV ID Kit easy to use within my laboratory.’

	Strongly Disagree	Disagree	Neutral	Agree	Strongly agree
CMR A	□	□	□	■	□
CMR B	□	□	□	□	■
CMR C	□	□	□	■	□
GEO A	□	□	□	■	□
GEO B	□	□	□	■	□

3. ‘I found all elements of the testing procedure easy to carry out’ Choose an item.

	Strongly Disagree	Disagree	Neutral	Agree	Strongly agree
CMR A	■	□	□	□	□
CMR B	□	□	□	□	■
CMR C	□	□	□	■	□
GEO A	□	□	■	□	□
GEO B	□	□	■	□	□

4. ‘I did not experience any technical problems when using the equipment’ Choose an item.

	Strongly Disagree	Disagree	Neutral	Agree	Strongly agree
CMR A	□	■	□	□	□
CMR B	□	■	□	□	□
CMR C	□	■	□	□	□
GEO A	□	■	□	□	□
GEO B	□	■	□	□	□

If you answered 1 – Strongly Disagree or 2 Disagree, how were these dealt with?

- *Indeterminate retest or control failed retest (Georgia)*
- *When we print results there are two labels coming out*
- *Power cord for the printers are not holding very well, sometimes we have to hold them before printing*
- *We had the labels rolling on itself when printing and it was not easy to remove*
- *It is a bit fastidious to fix the canal on the cartridge, it need a bit of physical effort*

- *We had issue with machine 02 that sometimes didn't recognize the printer, sometimes it sees the printer but refuse to print*

5. 'I found the on board Genedrive® software easy to navigate and interpret' Choose an item.

	Strongly Disagree	Disagree	Neutral	Agree	Strongly agree
CMR A	☐	☐	■	☐	☐
CMR B	☐	☐	☐	■	☐
CMR C	☐	☐	☐	■	☐
GEO A	☐	☐	☐	■	☐
GEO B	☐	☐	☐	■	☐

6. 'The packaging for the Genedrive® HCV ID Kit was fit for purpose' Choose an item.

	Strongly Disagree	Disagree	Neutral	Agree	Strongly agree
CMR A	☐	☐	☐	■	☐
CMR B	☐	☐	☐	■	☐
CMR C	☐	☐	☐	■	☐
GEO A	☐	☐	☐	☐	■
GEO B	■	☐	☐	☐	☐

If you answered 1 – Strongly Disagree or 2 – Agree, how do you think the packaging could be improved?

7. 'I would continue to use Genedrive® within my laboratory for HCV testing'

	Strongly Disagree	Disagree	Neutral	Agree	Strongly agree
CMR A	☐	☐	☐	■	☐
CMR B	☐	☐	☐	■	☐
CMR C	☐	☐	☐	■	☐
GEO A	☐	☐	☐	■	☐
GEO B	☐	☐	☐	■	☐

If agree or strongly agree, why?

- The system is less cumbersome and the method is well design and easy to learn
- The system for me doesn't have to be connected to a computer, it works very well and the screen is well readable. If we could launch several tests at the same time on one machine, it would be good as the reading time for one sample is relatively long
- A training is really necessary before using the machine, it is true the instruction is clear but technical assistance is required for the first use.

8. It's effective and straightforward, as well as convenient to use just one machine that can connect directly to the computer.

	Strongly Disagree	Disagree	Neutral	Agree	Strongly agree
CMR A	☐	☐	☐	☐	■
CMR B	☐	☐	☐	■	☐
CMR C	☐	☐	☐	☐	■
GEO A	☐	☐	☐	■	☐
GEO B	☐	☐	☐	■	☐

Questionnaire part 2: from https://www.usability.gov/

1. I think that I would like to use this system frequently.

	Strongly Disagree	Disagree	Neutral	Agree	Strongly agree
CMR A	□	□	■	□	□
CMR B	□	□	□	□	■
CMR C	□	□	□	■	□
GEO A	□	□	■	□	□
GEO B	□	□	■	□	□

2. I found the system unnecessarily complex.

	Strongly Disagree	Disagree	Neutral	Agree	Strongly agree
CMR A	□	■	□	□	□
CMR B	■	□	□	□	□
CMR C	□	■	□	□	□
GEO A	□	■	□	□	□
GEO B	□	□	□	■	□

3. I thought the system was easy to use.

	Strongly Disagree	Disagree	Neutral	Agree	Strongly agree
CMR A	□	□	□	□	■
CMR B	□	□	□	□	■
CMR C	□	□	□	□	■
GEO A	□	□	□	■	□
GEO B	□	□	□	■	□

4. I think that I would need the support of a technical person to be able to use this system.

	Strongly Disagree	Disagree	Neutral	Agree	Strongly agree
CMR A	■	□	□	□	□
CMR B	□	■	□	□	□
CMR C	□	■	□	□	□
GEO A	□	□	□	■	□
GEO B	□	□	□	■	□

5. I found the various functions in this system were well integrated.

	Strongly Disagree	Disagree	Neutral	Agree	Strongly agree
CMR A	□	■	□	□	□
CMR B	□	□	□	■	□
CMR C	□	■	□	□	□
GEO A	□	□	□	■	□
GEO B	□	□	□	■	□

6. I thought there was too much inconsistency in this system.

Strongly Disagree Disagree Neutral Agree Strongly agree

CMR A	□	■	□	□	□
CMR B	□	■	□	□	□
CMR C	□	■	□	□	□
GEO A	□	■	□	□	□
GEO B	□	■	□	□	□

7. I would imagine that most people would learn to use this system very quickly.

	Strongly Disagree	Disagree	Neutral	Agree	Strongly agree
CMR A	□	□	□	■	□
CMR B	□	□	□	■	□
CMR C	□	□	□	■	□
GEO A	□	□	□	□	■
GEO B	□	□	□	□	■

8. I found the system very cumbersome to use.

	Strongly Disagree	Disagree	Neutral	Agree	Strongly agree
CMR A	■	□	□	□	□
CMR B	■	□	□	□	□
CMR C	■	□	□	□	□
GEO A	■	□	□	□	□
GEO B	■	□	□	□	□

9. I felt very confident using the system.

	Strongly Disagree	Disagree	Neutral	Agree	Strongly agree
CMR A	□	□	□	■	□
CMR B	□	□	□	■	□
CMR C	□	□	□	■	□
GEO A	□	□	□	■	□
GEO B	□	□	□	■	□

10. I needed to learn a lot of things before I could get going with this system.

	Strongly Disagree	Disagree	Neutral	Agree	Strongly agree
CMR A	□	□	□	■	□
CMR B	□	□	□	■	□
CMR C	□	□	□	■	□
GEO A	□	□	■	□	□
GEO B	□	□	■	□	□
